# Social behaviour change interventions in eye care: lessons from the field

**Published:** 2022-09-20

**Authors:** Sumrana Yasmin, Nazaradden Ibrahim

**Affiliations:** Deputy Technical Director, Eye Health and URE: Sightsavers, Islamabad, Pakistan.; Global Technical Lead, Eye Health (West Africa): Sightsavers, Abuja, Nigeria.

**Social behaviour change interventions can help to increase the demand for eye health services and encourage communities to seek eye care.**

**Figure:**
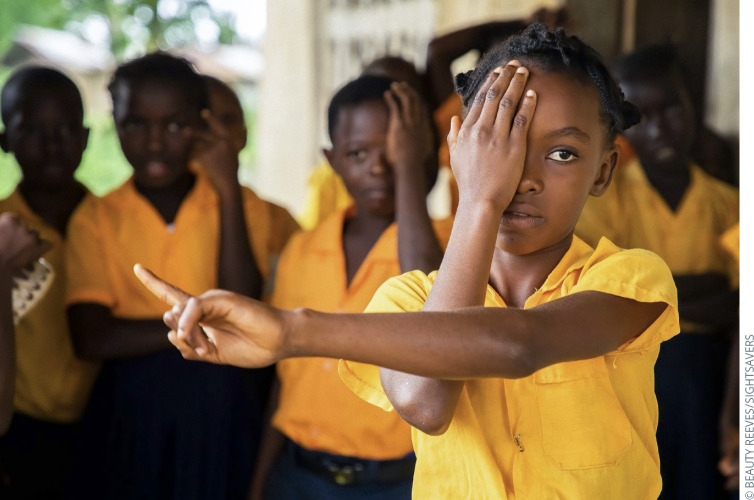
Improved access to school vision screening following community-based social behaviour change activities. liberia

Social behaviour change (SBC) is about understanding and influencing healthy and inclusive behaviours and providing a supportive social environment in which these behaviours can flourish. If we can encourage people to change their everyday behaviours, then we might get some way towards having healthier and more inclusive societies.

Supporting individuals and wider communities to make meaningful, long-term changes to their behaviour, including how they look after their eye health and how (and when) they seek eye care, is vital if we are to achieve the World Health Organization's Sustainable Development Goals.^1^

People's behaviour is influenced by many factors, including gender, age, disability, and personal beliefs (at the individual level); family income, class, religion, and location (at the family level); cultural practices and beliefs (at the community level), and the political situation (at the community or national level).

As we saw in the recent *Community Eye Health Journal* issue on primary eye health care (**bit.ly/DeliverEye**), health promotion – actions that bring about better eye health – involves both improving the knowledge, attitudes, and behaviour of individuals, and influencing the physical, cultural, and policy environment needed to support health and health-seeking behaviour.

Behaviour change interventions can be instrumental in improving access to, and the use of, eye care services. It is therefore vital to invest more in health promotion and education. It is equally important to review the impact of embedding behaviour change strategies in eye health programmes and to identify any lessons learnt.

## The steps of a behaviour change intervention

Planning a behaviour change intervention starts with answering the following questions.

- What behaviour do we want to change or promote?
- What supporting behaviour will be needed?
- What do we know about the behaviour and our target audience?
- What are the gaps in knowledge on how to create change?
- What needs to change for the behaviour to be practised?
- How do we create the change? (intervention categories and materials)
- What outcome do we want to achieve?

Table 1 gives a worked example, with the objective of increasing school attendance of girls with disabilities aged 6–14 years.

**Table 1:** An example of a social behaviour change plan using the six lessons from the project.

**Project objective**	**What behaviour do we want to change or promote?**	**Supporting behaviour**	**What is known about current behaviour and our target audience(s)?**	**What are the gaps in our knowledge on how to create change?**	**What needs to change for the behaviour to be practised?**	**How to create the change (Intervention categories and materials)**	**Project outcome**
Increase school attendance of girls with disabilities aged 6–14 years	Girls with disabilities aged 6–14 should attend school every day during the school term	Parents must make sure their children with disabilities attend school every day	Mothers currently prioritise their daughters’ housework over school attendance Children with disabilities face stigma and bullying in school, which discourages them from attending	We need to understand the community's cultural beliefs, and how to leverage or challenge them What are the most effective ways to motivate parents? How do we create a new norm in the school to reduce stigma and bullying?	Establish school attendance for children with disabilities as an aspiration and social norm for parents Children with disabilities should not experience bullying and negative language in the school environment	Community interpersonal communications are key for shifting cultural beliefs and perceptions about disability at the school A persuasive communication campaign aimed at children should be developed	Improved school attendance of girls with disabilities, resulting in increased participation and better education outcomes

The social behaviour change plan example and useful tips are based on the Sightsavers’ Social Behaviour Change Toolkit. You can learn more about Sightsavers’ social behaviour change learning journey at **https://bit.ly/3ezeB5y**

## Focus points for behaviour change interventions

Lessons from across a range of eye health programmes supported by Sightsavers indicate that the success of behaviour change interventions relies on following a robust approach that focuses on the following:

1. **Clarity.** There must be a focus on specific behaviours and audiences. We need to remember that people will not be able to change too many behaviours all at once, and what works for one group might not work for another. Experience has shown that identifying target audiences and the specific behaviours to be encouraged or discouraged is a key step to designing an effective behaviour change programme. Audiences could be children, women, teachers, or community members, depending on the planned project or service.
2. **Structure.** There needs to be a step-by-step process to analyse, design, deliver, and monitor behaviour change interventions. A project and communications plan should identify the issues to be addressed; changes that are desired; the audience to be targeted; key messages and channels of message delivery; and the proposed solutions.
3. **Evidence-based analysis.** Early analysis is important to avoid assumptions and understand what will motivate communities to change. This needs to be an engaging and collaborative process. It is also important to draw on the existing evidence base to find out what behaviour change strategies could work in particular settings.
4. **Community participation.** It is important to make sure that planned activities are piloted and suitable for local audiences, which should include all stakeholders, including people with disabilities. Messages, materials, and visuals must be suitable for the local context, preferably using local imagery, language, and design.
5. **Gender- and disability-inclusion.** It is important to develop behaviour change messages and visuals that are clear, accessible, and inclusive of all groups in society who might be excluded or have less influence in that context: e.g., women, people with disabilities, and nomadic groups.
6. **Innovation and technology.** Technology platforms, including mobile health (mHealth) and social media, can be useful to reach a broader audience. The messaging needs to be cohesive and succinct to ensure consistency and long-term impact.
7. **Regular and consistent communication.** A mix of different channels – such as face-to-face interactions, community radio, and community events – can increase the likelihood of communications resulting in the desired change. For maximum impact, it is important to:
  - use plain and straightforward language
  - create simple, brief, clear, and accessible messages to convey core concepts
  - combine written text with visual information so it can be understood by all.
8. **Community influencers and role models.** Women's groups, religious leaders, and organisations of persons with disabilities can be co-opted to serve as role models and motivators for behavioural change.

## An integrated school health programme

Lack of adherence to treatment and to the use of spectacles is a common problem in many low- and middle-income countries, which is often the result of lack of knowledge of eye health as well as negative perceptions, attitudes, and misconceptions about wearing spectacles.^1^

Using a targeted social and behaviour change approach, the school health integrated programme in Liberia, implemented between 2019-2021, aimed to provide vision screening to 76,000 children of school age. However, nearly 123,000 children were screened (far more than expected) and 590 received corrective glasses. This outcome was achieved by clearly identifying our target audience and the behaviour we needed to address, and by using local content and approaches. The lessons from Sightsavers’ school health integrated programme in Liberia, and a similar programme in Pakistan, showed that understanding why girls and boys wear (or do not wear) spectacles will help us to design effective behaviour change interventions that address barriers to their use. Some of the common barriers include:

- lack of awareness
- negative perceptions
- traditional beliefs about the use of spectacles, especially by girls
- distance
- cost.

Developing appropriate information and communication materials to target the barriers will go a long way in creating awareness about eye health services and products.

Gaining community support by engaging with parents (through parent–teacher associations) and with traditional and religious leaders may help to address misconceptions and traditional beliefs.

Different operational strategies should be employed to address the problem of distance to eye care services. For example, to reduce the distance that children may have to travel for refraction and spectacles, a central location can be designated for eye health services within a cluster of schools.

Cost recovery mechanisms can be put in place to ensure that services and spectacles are affordable for everyone.

Considering the large population of out-of-school children in low- and/or middle-income countries, these programmes also sought to identify the best approaches for encouraging both enrolled and non-enrolled school-aged children to participate in vision screening and deworming activities. Approaches included the use of peer groups (mostly made up of enrolled children who had been screened and received their deworming tablets) to encourage non-enrolled children to access the service. Engagement with community-based organisations, community volunteers, and town criers can also helped to spread information.

**Figure:**
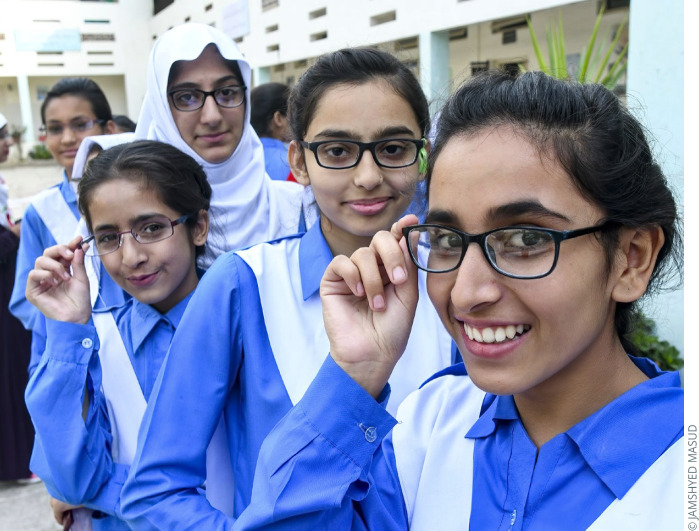
Social behaviour change interventions focused on ensuring adherence to the use of spectacles at at a school in Pakistan. pakistan
